# Supplementary material for: A Novel Triplet of Alisertib Plus Ibrutinib Plus Rituximab Is Active in Mantle Cell Lymphoma
Source: Cancers (Basel). 2024 Dec 21;16(24):4257. doi: 10.3390/cancers16244257 (PMC11674227; doi:10.3390/cancers16244257)
Supplement: Supplementary file 1 [file cancers-16-04257-s001.zip › cancers-3228063-supplementary.pdf]

“A Novel Triplet of Alisertib Plus Ibrutinib Plus Rituximab Is Active in Mantle Cell Lymphoma”

Baskaran Subramani [1], Patrick J. Conway [1,2], Aisha Al-Khinji [1,3], Kun Zhang [1], Ritu Pandey [4] and Daruka Mahadevan[1,2,3,4,*]

[1] Division of Hematology/Oncology, Department of Medicine, Mays Cancer Center, University of Texas Health San Antonio, San Antonio, TX 78229, USA; subramani@uthscsa.edu (B.S.); patrick.conway@keiseruniversity.edu (P.J.C.); aalkhinji@arizona.edu (A.A.-K.); zhangk@uthscsa.edu (K.Z.)
[2] Graduate School of Biomedical Sciences, University of Texas Health San Antonio, San Antonio, TX 78229, USA
[3] Clinical Translational Science Program, University of Arizona, Tucson, AZ 85721, USA
[4] Department of Cellular and Molecular Medicine, University of Arizona Cancer Center, Tucson, AZ 85721, USA; ritu@arizona.edu
* Correspondence: mahadevand@uthscsa.edu

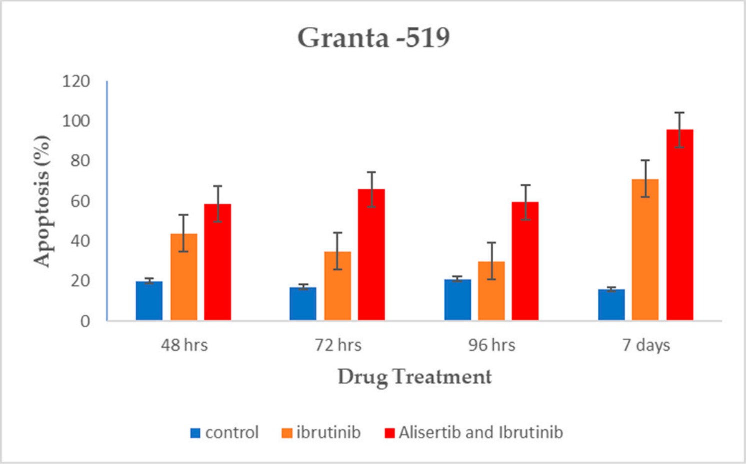

Figure S1: Apoptosis analysis was performed on Granta-519 cells treated with DMSO, Ibrutinib and combination of Alisertib and Ibrutinib for 48 hrs, 72 hrs, 96 hrs and 7 days.

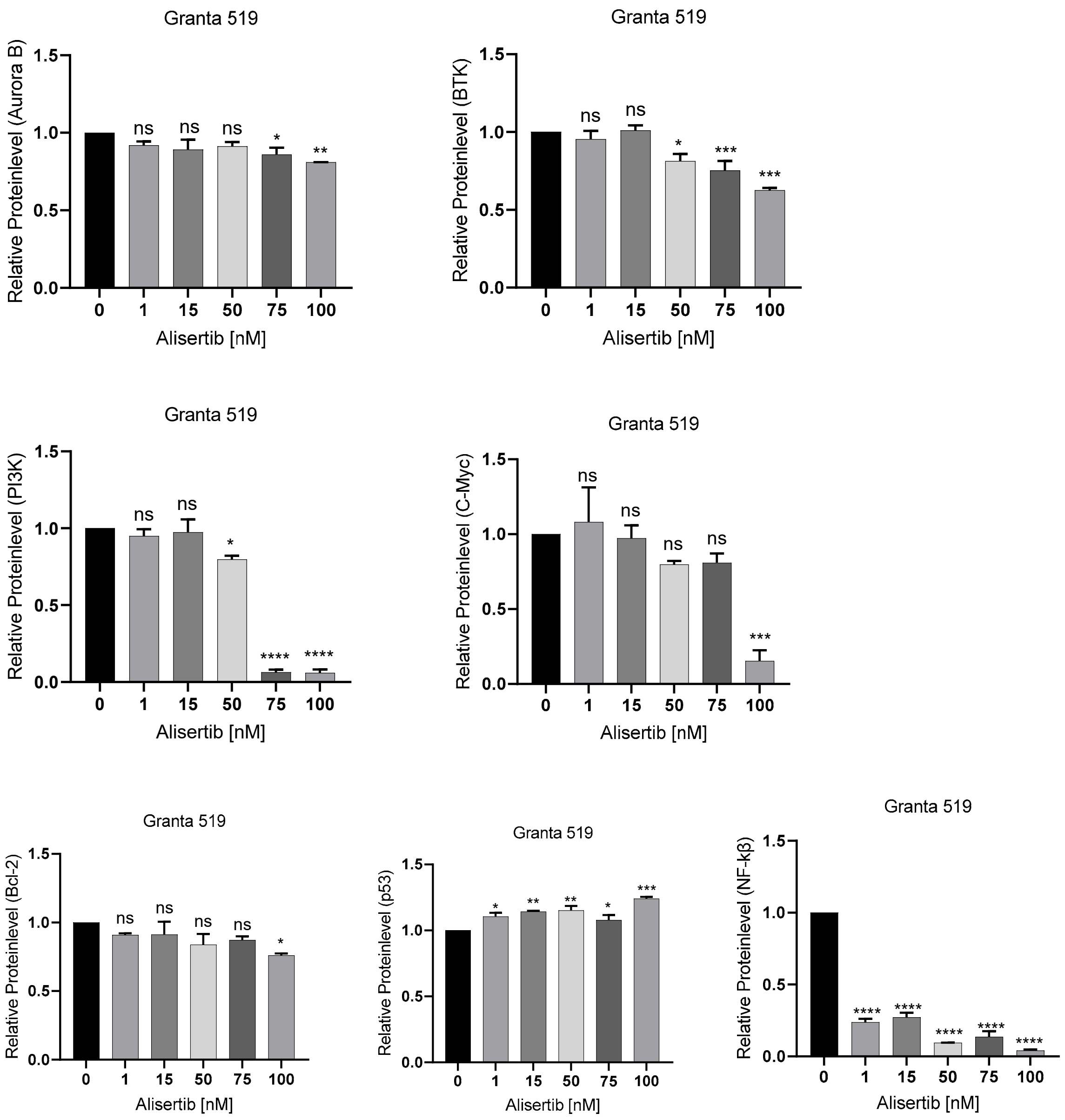

Figure S2: (A) Granta-519 cells (DMSO (0), 1, 25, 50, 75, 100 nM) the quantification of signal intensity of the blots. * p < 0.05; ** p < 0.01; *** p < 0.001; **** p < 0.0001; ns, not significant.

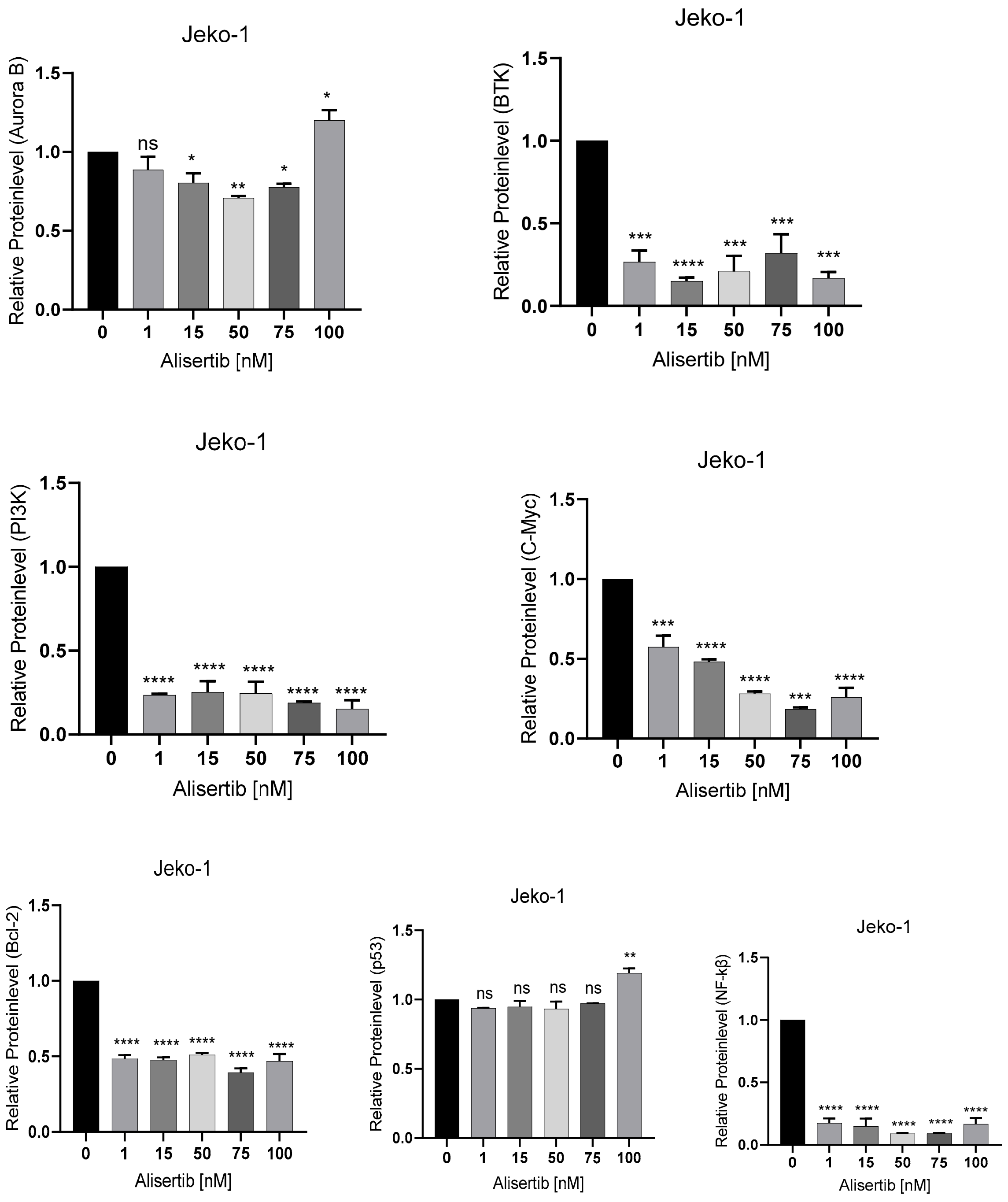

Figure S2: (B) Jeko-1 cells (DMSO (0), 1, 25, 50, 75, 100 nM) the quantification of signal intensity of the blots. * p < 0.05; ** p < 0.01; *** p < 0.001; **** p < 0.0001; ns, not significant.

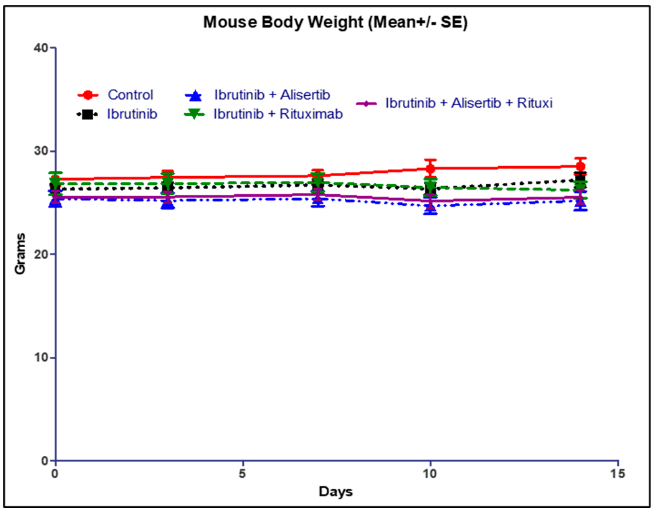

Figure S3: The body weights of the mice by control and treatment arms.

Table S1. Synergistic combinatory effect of alisertib with Ibrutinib. (A) Grants 519 Cells and (B) jeko-1 cells were treated with varying concentrations of alisertib + ibrutinib. CI values between 0.1–0.3 shows strong synergism.

A) Granta 519 cells		
alisertib (μM)	Ibrutinib (μM)	CI Values
0.001	0.001	0.791
0.003	0.003	0.732
0.006	0.006	0.695
0.012	0.012	0.473
0.025	0.025	0.332
0.05	0.05	0.323
0.1	0.1	0.274

B) Jeko-1 cells		
alisertib (μM)	Ibrutinib (μM)	CI Values
0.00061	9.38E-05	0.841
0.001221	0.000188	0.832
0.002441	0.000375	0.764
0.004883	0.00075	0.681
0.009766	0.0015	0.592
0.019531	0.003	0.241
0.039063	0.006	0.220
0.078125	0.012	0.226
0.15625	0.024	0.212

Granta-519 cells

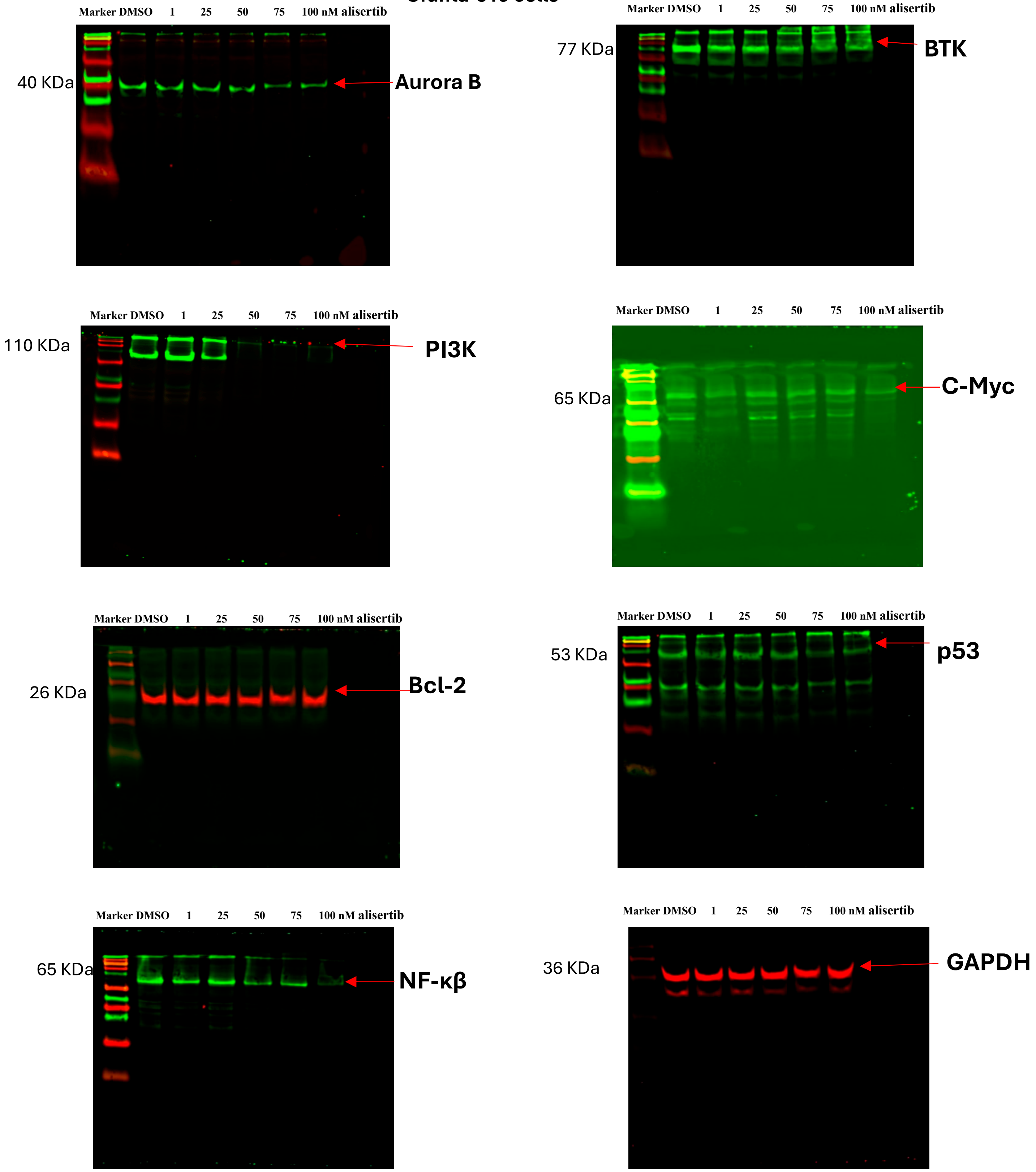

JeKo-1 cells

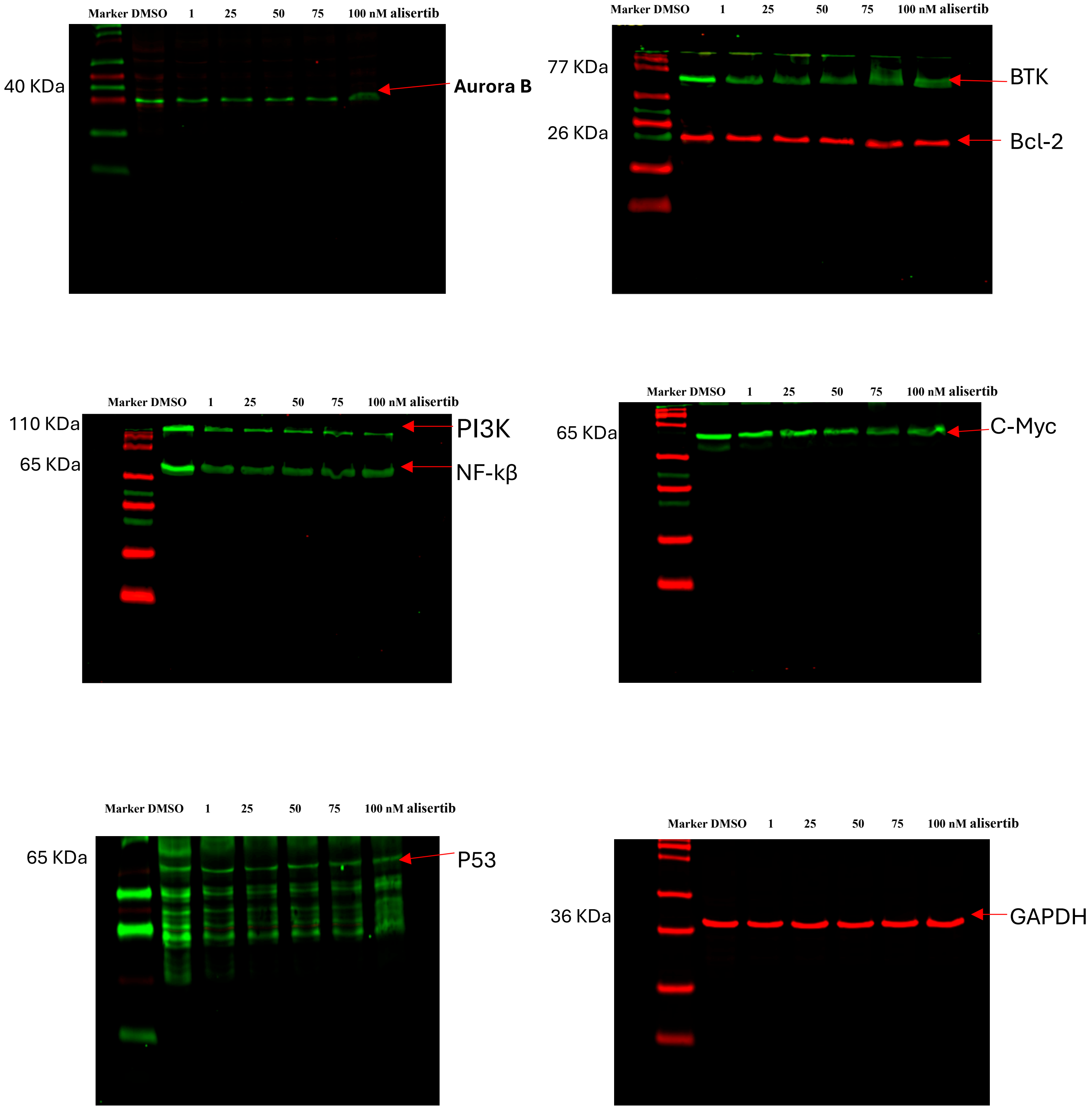

Figure S4: Uncropped Western Blots images.
